# Pharmacological characterization of linaprazan glurate (X842), a novel potassium-competitive acid blocker, *in vitro* and *in vivo*

**DOI:** 10.3389/fphar.2025.1636523

**Published:** 2025-09-05

**Authors:** Ming Lu, Yi Cui, Nailin Li, Yan He, Ling Zhou, Jin Xiu, Pingsheng Hu

**Affiliations:** ^1^ Sinorda Pharmaceuticals Ltd., Jing Yang Hi-Tech Park, Guiyang, Guizhou, China; ^2^ Clinical Research Center, The Affiliated Hospital of Guizhou Medical University, Guiyang, Guizhou, China; ^3^ Department of Medicine-Solna, Clinical Pharmacology Group, Karolinska University Hospital-Solna, Karolinska Institutet, Stockholm, Sweden; ^4^ Good Clinical Practice Center, The Affiliated Hospital of Guizhou Medical University, Guiyang, Guizhou, China; ^5^ Department of Generic, Department of Neurobiology, Care Sciences and Society, Division of Clinical Geriatrics, Karolinska Institutet, Stockholm, Sweden

**Keywords:** acid blocker, ATPase, competitive, gastric acid, potassium

## Abstract

**Introduction:**

The aim of this study is to characterize the pharmacology of linaprazan glurate (X842), an ethyl ester prodrug of linaprazan (a novel potassium-competitive acid blocker), in animal species *in vitro* and *in vivo* to achieve a better pharmacological profile.

**Methods:**

Pharmacokinetic profiling, hydrogen (H^+^)/potassium (K^+^)-ATPase inhibition, and gastric acid inhibition experiments were performed.

**Results:**

X842 was rapidly absorbed with a very low plasma concentration. X842 was rapidly transformed by enzymatic cleavage into its active metabolite, linaprazan, as shown by the half-life, maximum concentration, and area under the concentration–time curve of the two substances. Selective inhibition of the gastric H^+^/K^+^-ATPase and acid formation *in vitro* was observed. Linaprazan, X842, and vonoprazan selectively inhibited acid formation from gastric H^+^/K^+^-ATPase in a potassium-dependent manner. The inhibitory potency rank was vonoprazan > linaprazan > X842, with half-maximal inhibitory concentration (IC_50_) values of 17, 40, and 436 nM, respectively, showing that X842 is a very weak inhibitor of H^+^/K^+^-ATPase *in vitro*. In a pylorus-ligated rat model, X842 potently inhibited gastric acid secretion in a dose-dependent manner with a long duration of action, highlighting the two stages of pharmacokinetics that give the compound both its fast onset and its long-lasting duration of action on H^+^/K^+^-ATPase.

**Discussion:**

X842 is a prodrug that exerts pharmacological effects both independently and through its metabolized active compound linaprazan, with both a fast onset and a long duration of action. X842 could be a potential drug candidate worthy of further clinical study.

## 1 Introduction

Disorders of gastric acid production are a major pathological cause of gastroesophageal reflux disease (GERD) and peptic ulcers (Olbe et al., 2003). Therefore, control of gastric acid secretion has long been a goal in the management of such diseases. Since the 1980s, the most prescribed gastric acid secretion-suppressing drugs have been proton pump inhibitors (PPIs), which inhibit the proton exchange activity of the hydrogen (H^+^)/potassium (K^+^)-ATPase in the acid-secreting parietal cells of the stomach (Lindberg et al., 1990). In the treatment of *Helicobacter pylori* infection, PPIs are often used in combination with antibiotics, and esomeprazole is one of the commonly chosen PPIs (Malfertheiner et al., 2022). However, PPIs have shown some limitations in the treatment of severe GERD, including their slow onset of action and instability (Ichikawa et al., 2016; Fass, 2007). These limitations highlight the need for new acid-suppressing agents for GERD.

**FIGURE 1:**
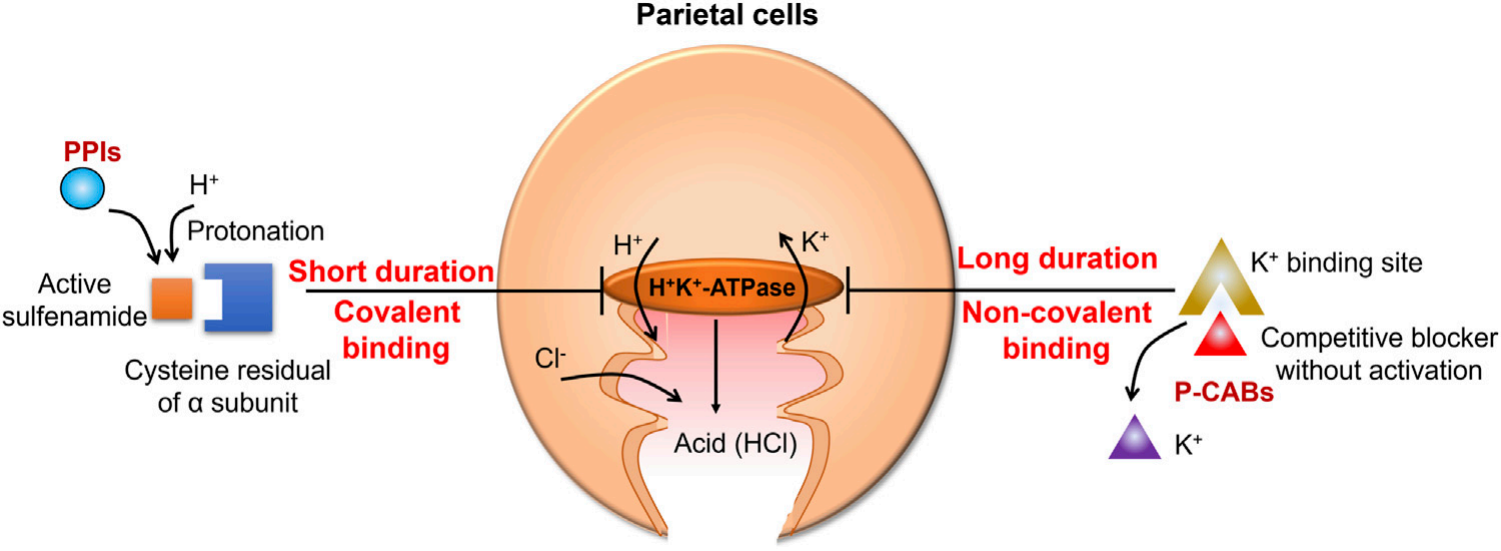
Mechanism of action of gastric acid secretion inhibitors. Proton pump inhibitors (PPIs) are converted to sulfenic acids and/or sulfenamides by protonation, irreversibly inhibiting ATPase. Potassium-competitive acid blockers (P-CABs) reversibly block the potassium-binding site of the hydrogen (H^+^)/potassium (K^+^)-ATPase and competitively inhibit proton transport with a fast onset and a prolonged duration of action.

Potassium-competitive acid blockers (P-CABs) are H^+^/K^+^-ATPase inhibitors that were developed in the 1980s (Oshima and Miwa, 2018). P-CABs stably accumulate in the acidic secretory canaliculus and inhibit acid secretion by non-covalently binding to H^+^/K^+^-ATPase (Figure 1) (Shin et al., 2011). This non-covalent binding means that P-CABs confer several advantages over PPIs, which bind covalently. Notably, P-CABs have a very slow dissociation rate and inhibit newly synthesized or newly inserted H^+^/K^+^-ATPase. In addition, PPIs have a slow onset of action, whereas P-CABs exert rapid effects (Andersson and Carlsson, 2005; Scott et al., 2016). Therefore, efforts have been made to further synthesize and characterize P-CABs, including imidazopyridine, pyrimidine, and pyrrole derivatives (Kondo et al., 2012; Inatomi et al., 2016). However, a limited number of P-CABs have been approved for clinical use (Kim et al., 2018; Wong et al., 2022; Leowattana and Leowattana, 2022; Blair, 2024). Therefore, there is still a need for additional P-CABs to address the unmet clinical needs, improve efficacy, and reduce toxicity (Agago et al., 2024).

Linaprazan is a novel P-CAB developed by AstraZeneca. It is a reversible inhibitor of H^+^/K^+^-ATPase with high selectivity, and its pharmacological and toxicological effects have been well characterized *in vitro* and *in vivo*. Although the inhibitory effect of linaprazan on acid secretion can last for 24 h in rats, linaprazan is relatively short-acting in humans (Gedda et al., 2007; Scarpignato and Hunt, 2024). Moreover, phase II clinical studies have demonstrated no superior efficacy compared with esomeprazole in terms of esophagitis healing (Dent et al., 2008). To improve its pharmacological properties, the structure of linaprazan was modified by esterification with glutaric acid. After screening the modified compounds, X842 was selected for further pharmacological studies, both *in vitro* and *in vivo*, using animal models. The pharmacological data of X842 are reported herein.

## 2 Materials and methods

### 2.1 Ethics

The study protocol was approved by the Institutional Animal Care and Use Committee of Shanghai Medicilon Inc (ethics approval numbers MED1405_P001, MED1405_P003, 10012-14007, and 10012-14008).

### 2.2 Pharmacokinetic assessment

#### 2.2.1 Rat experiments

Male and female Sprague-Dawley rats weighing approximately 150 g were housed individually with a 12-h light/dark cycle. Food and water were provided *ad libitum*, except during overnight fasting (10–16 h) prior to oral drug administration, as described previously (Kotegawa et al., 1998; Kotegawa et al., 2002). To investigate the absorption kinetics of X842 (prodrug) and linaprazan (metabolite, parent drug) in rats after oral and intravenous administration of X842, X842 were dissolved in 5% Solutol HS15/45% ethanol/10% PEG400/40% saline, and methane sulfonic acid was used to adjust to pH 3, yielding solutions with concentrations of 0.6 mg/mL, 2.4 mg/mL, and 9.6 mg/mL for oral administration. For intravenous (IV) injection, a drug solution (0.6 mg/mL) was prepared and filtered through a 0.22-μm filter. Blood samples were collected via the jugular vein of conscious rats (without intubation, narcotics, or antibiotics) prior to dosing and at 0.083, 0.25, 0.5, 1, 2, 4, 6, 8, and 24 h after dosing. Blood samples were placed into tubes containing sodium heparin and centrifuged at 3,500 rpm for 10 min at 4 °C. Following centrifugation, the plasma was transferred to clean tubes and stored at −80 °C until further analysis. All animals were euthanized by carbon dioxide (CO_2_) inhalation after the final blood sample collection.

Pharmacokinetic assessments were conducted by Shanghai Medicilon Inc. Using C_18_ solid-phase extraction (Shimadzu UFLC-20A, ACE 3, C_18_, 3.0 µm, 150 × 4.6 mm; Shimadzu, Japan) with the following conditions: mobile phase A: 1 M ammonium acetate in water, mobile phase B: 100% acetonitrile; gradient 0–5 min: 100% A, 5–30 min: 85% A, 35–63 min: 75% A, 63–65 min: 55% A, 65–70 min: 0% A, 72–87 min: 100% A; flow rate 0.7 mL/min; column temperature 30 °C (Weidof and Covey, 1992). A non-compartmental module of WinNonlin Professional v.6.2 (Pharsight, US) was used to calculate the pharmacokinetic parameters, including the area under the concentration–time curve [AUC(_0–24h_)], half-life (t_1/2_), and maximum concentration (C_max_). Bioavailability was calculated as F (%) = (Dose_iv_ × AUC_po_(_0–24h_)) ÷ (Dose_po_ × AUC_iv_ (_0–24h_)) × 100%, where “po” represents oral administration and “iv” represents intravenous administration.

#### 2.2.2 Canine experiments

Male and female Beagle dogs weighing around 9 kg were arbitrarily selected for testing. Food and water were provided *ad libitum*, except during overnight fasting (15–16 h) prior to oral drug administration (Adebowale et al., 2002). X842 were dissolved in the PEG400/ethanol/Solutol/water mixture (40:10:5:45% v/v) and methane sulfonic acid was used to adjust to pH 3, yielding solutions with concentrations of 0.3 mg/mL, 1.2 mg/mL, and 4.8 mg/mL for oral administration. There were four rounds of drug administration. One week was required between the rounds of testing according to the conditions for of animal welfare maintenance. For IV injection, a drug solution with a concentration of 0.3 mg/mL was prepared and filtered through a 0.22-μm filter. Blood samples were collected via the jugular vein using the same procedures as were used for the rat experiments. Following centrifugation, the plasma was transferred to clean tubes and stored at −80 °C until further analysis. All animals were euthanized by phenobarbital injection after the final blood sample collection. Pharmacokinetic assessments were conducted as described in 2.2.1.

### 2.3 Pharmacodynamic assessment

#### 2.3.1 Inhibition of H^+^/K^+^-ATPase activity

The H^+^/K^+^-ATPase inhibition reaction was performed in buffer (50 mM Tris-HCl, 5 mM magnesium chloride, 10 μM valinomycin, pH 6.5) with or without 20 mM potassium chloride (KCl), and inorganic phosphate (Pi) was detected using Malachite Green solution, made from 0.1% malachite green, 1.5% hexaammonium molybdate, and 0.2% Tween-20 in a ratio of 100:25:2 (Skou, 1982; Feng et al., 2011). Gastric H^+^/K^+^-ATPase was prepared from rabbit glands, as described previously (Kondo et al., 2012; Arikawa et al., 2012). The reaction occurred in a 96-well plate with a total volume of 50 µL/well, including buffer, enzyme, ATP, and drug. After 30 min at 37 °C for enzyme stability, ATP was added at 0.2 mM to start the reaction, which was optimized from the titration experiments. The reaction lasted 20 min at 37 °C, followed by the addition of Malachite Green for the colorimetric assay. The enzyme control wells contained the full enzyme system, while the buffer control wells contained only buffer to correct for background values. Absorbance was measured at 620 nm using a plate reader (SpectraMax M5). The inhibition rate was calculated using the following formula, accounting for the enzyme activity in the presence and absence of K^+^:

$$Inhibition\;Rate\;\left(\%\right)=\left(\frac{{\text{OD}}_{\text{with}\;K+\text{and}\;\text{enzyme},\text{without}\;\text{drug}}-{\text{OD}}_{\text{with}\;K+\text{and}\;\text{enzyme},\text{with}\;\text{drug}}\;}{{\text{OD}}_{\text{with}\;K+\text{and}\;\text{enzyme},\text{without}\;\text{drug}}-{\text{OD}}_{\text{without}\;K+\text{and}\;\text{enzyme},\text{without}\;\text{drug}}}\right)\times 100$$

where “OD with K^+^ and enzyme, without drug” is the optical density measured at 620 nm in the presence of K^+^ and enzyme, without the tested drug (enzyme control wells); “OD with K^+^ and enzyme, with drug” is the optical density measured at 620 nm in the presence of K^+^ and enzyme, with the tested drug; and “OD without K^+^ and enzyme, without drug” is the optical density measured at 620 nm in the absence of K^+^ and enzyme, without the tested drug (buffer control wells).

#### 2.3.2 Gastric acid inhibition in pylorus-ligated rats

The rats were fasted overnight with *ad libitum* access to water. Drugs and vehicle were administered orally to rats in a blinded manner. The pylorus was ligated under ether anesthesia within 1 h after drug and vehicle administration, and the abdomen was closed by suturing. At 4 h after pyloric ligation, the rats were sacrificed by CO_2_ asphyxiation, and the stomach was removed (Hori et al., 2010). The gastric contents were collected and centrifuged at 3,000 rpm for 10 min. The volume of each sample was measured, the gastric acid concentration was determined by acid-base titration, and the total acidity was calculated.

### 2.4 Statistical analysis

The data are reported as the mean ± standard deviation. The dose–response curves and the half-maximal inhibitory concentration (IC_50_) were determined by non-linear regression using GraphPad Prism 8.0. All statistical analyses were performed using SPSS Statistics 21.0 software (SPSS Korea, Seoul, Republic of Korea). *P* < 0.05 was considered statistically significant.

## 3 Results

### 3.1 Pharmacokinetic profile of X842 in rats

**FIGURE 2:**
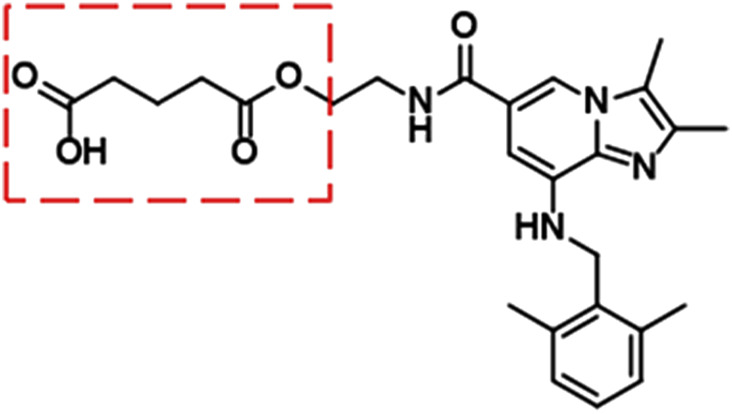
Chemical structure of X842. As a prodrug of linaprazan, X842 was esterified with glutaric acid and contained a free hydroxyl group.

**FIGURE 3:**
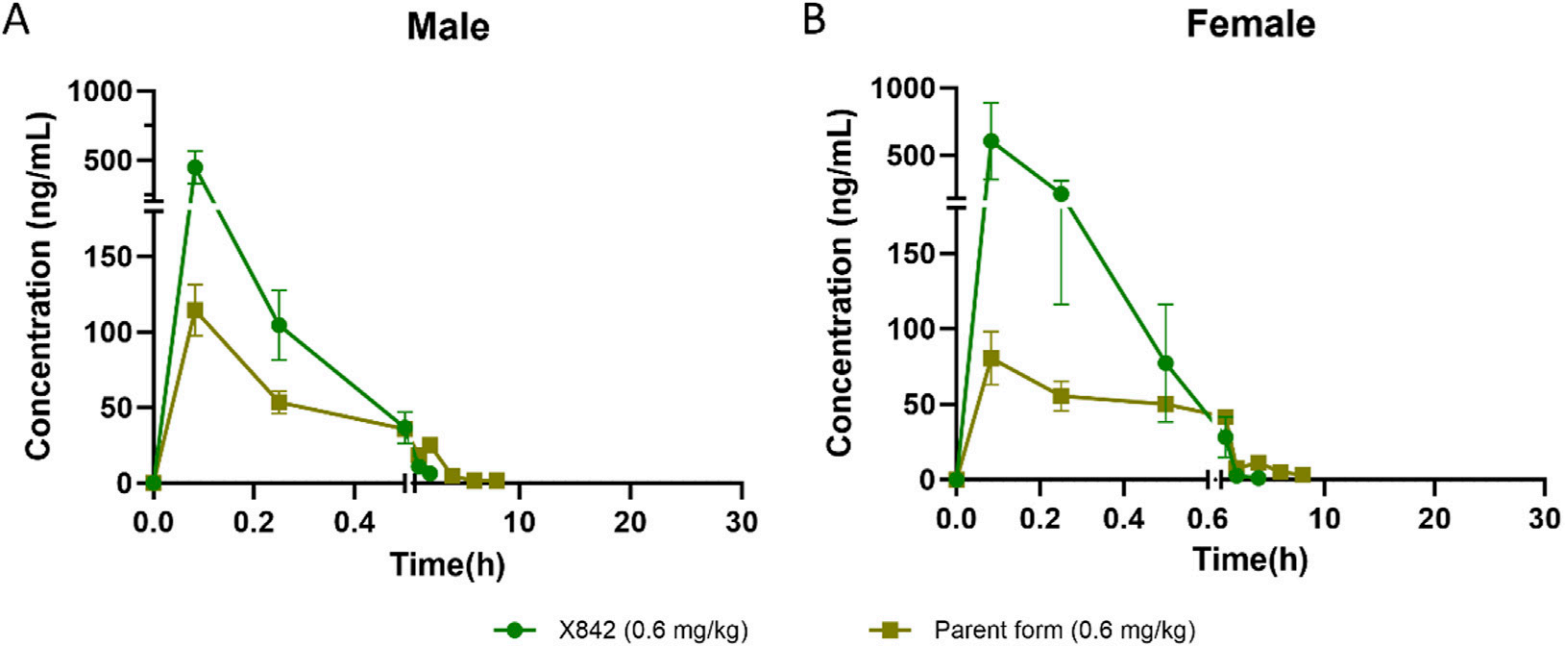
Plasma concentration of X842 after intravenous administration in male **(A)** and female **(B)** rats. Both the parent drug (linaprazan) and the prodrug (X842) were tested to evaluate their pharmacokinetic profiles (N = 6 per group).

**FIGURE 4:**
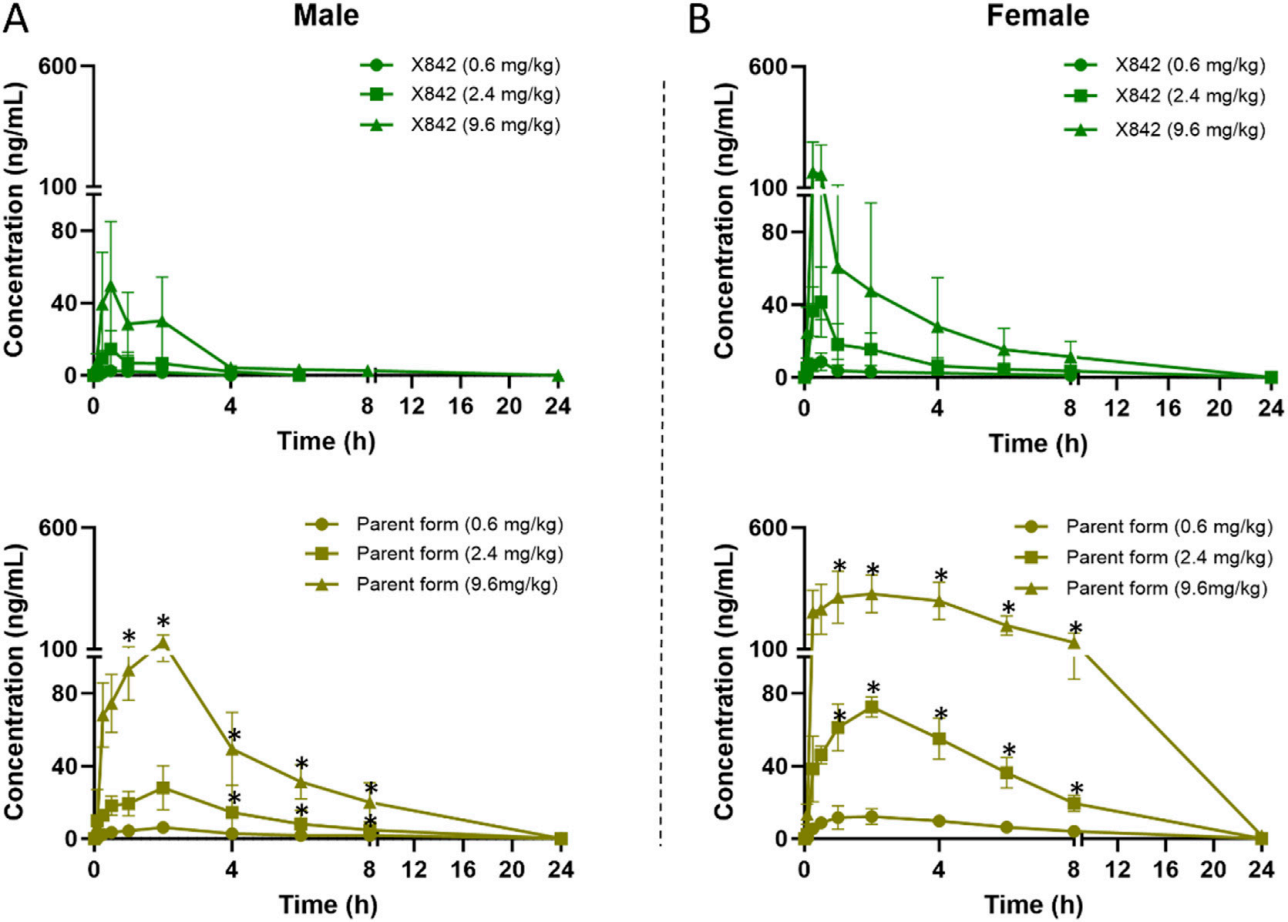
Plasma concentration of X842 after oral administration in male **(A)** and female **(B)** rats. Both the prodrug and the parent drug were evaluated (N = 6 per group). *P < 0.05, comparing the blood concentration of X842 with its metabolized parent drug at the same time points after oral administration. Supplementary Figure S2 shows a magnified view with visible error bars for the parent drug in the 0.6 mg/kg dose group.

**FIGURE 5:**
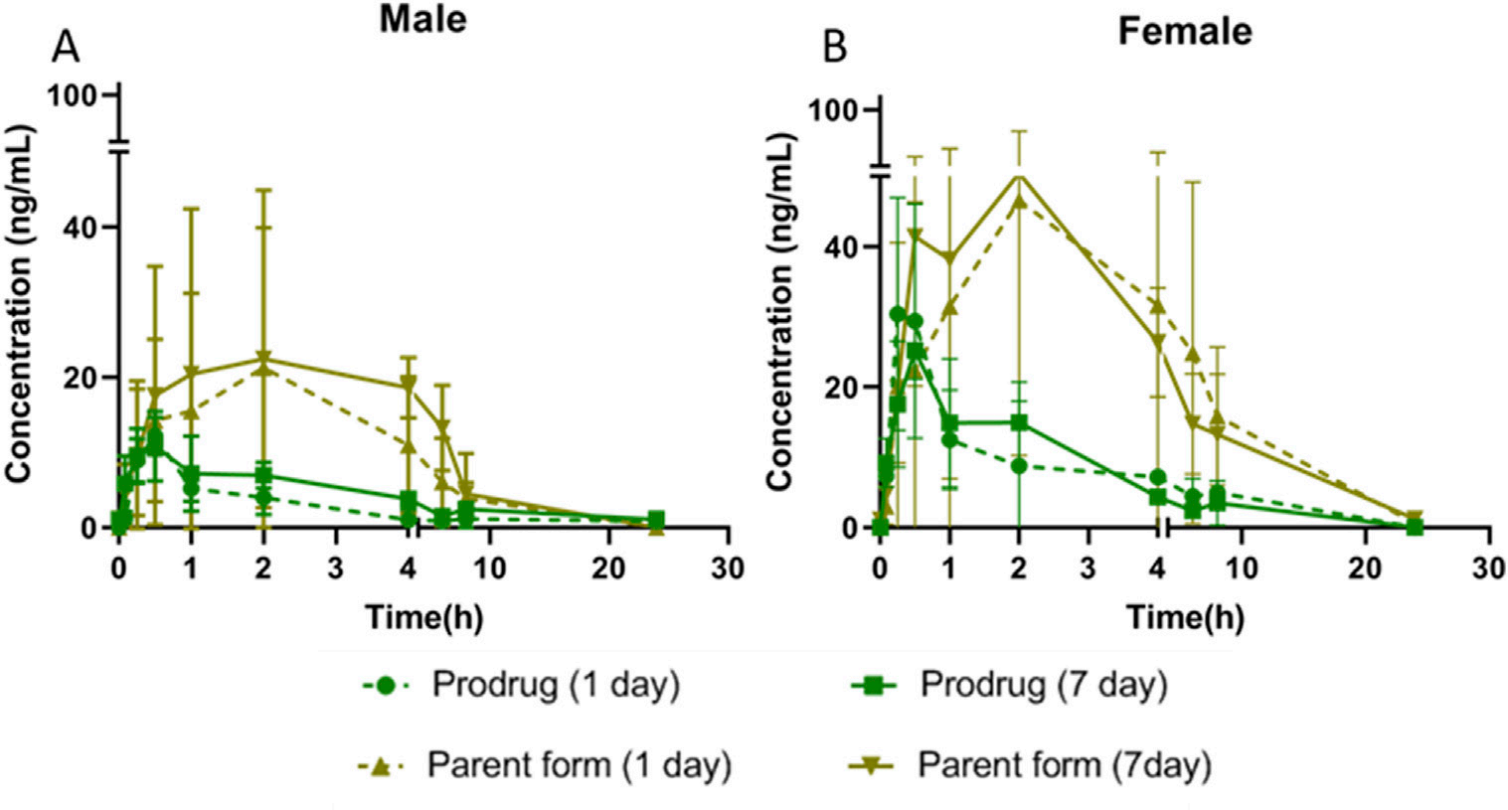
Plasma concentration of X842 on Day 1 and Day 7 after repeated oral administration in male **(A)** and female **(B)** rats (N = 3 per group).

X842 was designed as a linaprazan prodrug by side chain esterification with glutaric acid (Figure 2). We investigated the pharmacokinetic profile of X842 after IV and oral administration. To determine the biotransformation of X842 (prodrug) to linaprazan (parent drug), the pharmacokinetic characteristics of both compounds were evaluated. Sixty Sprague-Dawley rats (equal distribution of males and females) were randomly assigned to five groups. Group 1 underwent IV administration of the test drugs (Figure 3; Table 1). Groups 2, 3, and 4 received increasing concentrations of orally administered drugs (Figure 4; Table 2). Group 5 received repeated administrations of orally administered drugs for seven consecutive days (Figure 5; Table 3).

**TABLE 1:** Pharmacokinetic properties of X842 when administered intravenously in male and female rats.

Parameter		Male		Female	
		Prodrug	Parent	Prodrug	Parent
t_1/2_	h	0.4 ± 0.1	1.4 ± 0.1	0.4 ± 0.03	2.0 ± 0.4
C_max_	ng/mL	448.1 ± 119.9	114.5 ± 17.0	606.2 ± 284.5	80.5 ± 17.7
AUC_(0–24h)_	h*ng/mL	145.6 ± 38.0	114.2 ± 7.1	217.8 ± 101.0	133.2 ± 11.1

AUC_(0–24h)_, area under the concentration–time curve; C_max_, maximum concentration; t_1/2_, half-life. All animals received X842 only. The concentration of the parent drug reflects the *in vivo* metabolic conversion of linaprazan.

**TABLE 2:** Pharmacokinetic parameters of X842 after oral administration in male and female rats.

Parameter	Unit	0.6 mg/kg		2.4 mg/kg			9.6 mg/kg
		Prodrug	Parent	Prodrug	Parent	Prodrug	Parent
Male							
t_1/2_	h	NA	2.2 ± 1.0	2.6 ± NA	2.6 ± 0.5	2.01 ± 0.18	3.1 ± 0.3
C_max_	ng/mL	2.5 ± 1.8	6.2 ± 1.7	14.6 ± 10.1	32.0 ± 9.7	49.7 ± 35.4	128.1 ± 17.5^*^
AUC_(0–24h)_	h*ng/mL	2.5 ± 2.6	22.1 ± 9.9	18.3 ± 10.0	134.5 ± 43.3^*^	11.6 ± 77.5	575.8 ± 46.1^*^
Female							
t_1/2_	h	2.8 ± NA	3.7 ± 1.6	4.1 ± 1.2	2.7 ± 0.4	2.1 ± 0.3	2.6 ± 0.1
C_max_	ng/mL	8.7 ± 4.9	13.5 ± 5.8	42.6 ± 17.4	72.5 ± 5.6^*^	164.3 ± 123.9	336.4 ± 69.4^*^
AUC_(0–24h)_	h*ng/mL	13.8 ± 13.1	67.6 ± 23.7^*^	85.6 ± 45.6^*^	382.3 ± 44.9^*^	309.8 ± 255.9	3,022.3 ± 785.6^*^

*P < 0.05, compared with the same parameter in male rats (independent-samples t-test).

NA: little data below the limit of quantification were available, so parameters were not detectable.

AUC_(0–24h)_, area under the concentration–time curve; C_max_, maximum concentration; t_1/2_, half-life.

**TABLE 3:** X842 accumulation after 7 days of repeated oral administration in rats.

Dose (2.4 mg/kg)	AUC_(0–24 h)_			
	Day 1		Day 7	Accumulation coefficient
Prodrug form	Male	38.7 ± 33.8	71.1 ± 51.4	1.8^a^
	Female	93.7 ± 32.4	83.9 ± 25.1	0.9
Parent form	Male	144.1 ± 33.1	185.9 ± 57.6	1.3^a^
	Female	484.0 ± 221.3	433.2 ± 166.5	0.9

Accumulation coefficient = AUC_(0–24h)_ (Day 7 of repeat administration) ÷ AUC_(0–24h)_ (Day 1 of repeat administration).

^a^ An accumulation effect was observed, but it was not significantly different.

AUC_(0–24h),_ area under the concentration–time curve.

The plasma concentration of X842 was barely detectable within 0.5 h after IV administration in both male and female rats (Figure 3). This rapid decline in the X842 concentration indicated that X842 was quickly converted into its active metabolite, linaprazan, *in vivo*. For single-dose oral administration, t_1/2_ ranged from 2.0 to 2.7 h in male rats, and from 2.1 to 4.1 h in female rats (Table 2). Notably, the blood concentration of linaprazan was significantly higher than that of its prodrug X842 after 4 h when administered at doses of 2.4 mg/kg and 9.6 mg/kg (*P* < 0.05; Figure 4). This significant difference in the blood concentrations between the prodrug and its active metabolite is critical for understanding the pharmacokinetics of X842.

The plasma exposure calculation showed that the AUC_(0–24h)_ of the parent drug was 5.1–9.8 times that of the prodrug, indicating significantly greater overall exposure. Notably, the pharmacokinetic profile was consistent with the reported data for similar compounds, such as vonoprazan, which also demonstrates rapid absorption and significant conversion to active metabolites *in vivo* (Kong et al., 2020). Rapid conversion to the active metabolite ensures that the drug rapidly reaches its therapeutic concentration, which is essential for the effective treatment of acid-related diseases, such as GERD (Ashida et al., 2015).

Following single oral dosing, female rats consistently exhibited higher systemic exposure than males (Table 2). C_max_ and AUC_0–24h_ were significantly greater at every dose tested (*P* < 0.05), whereas no sex difference was seen after IV administration, indicating that absorption rather than clearance underlies the disparity. When the same doses were administered orally for seven consecutive days (Table 3), modest accumulation occurred in males (AUC _Day7_ ÷ AUC _Day1_ = 1.8 for the prodrug and 1.3 for the parent drug), but not in females (ratio = 0.9). This suggests that saturation or auto-inhibition of first-pass metabolism develops only in males upon repeated dosing. Consequently, the absolute oral bioavailability at 0.6 mg/kg (calculated from the combined exposure of parent drug plus active metabolites) was 11% in males versus 27% in females (Table 4), confirming both the sex-dependent absorption and the mild accumulation observed in males.

**TABLE 4:** Bioavailability calculation based on the sum of exposures of X842 (prodrug) and linaprazan (parent drug).

Sex (0.6 mg/kg)	AUC_(0–24h)_ (h*nmol/L)	AUC_(0–24h)_ (h*nmol/L)	Bioavailability
	po	iv	%
Male	65.6	595.8	11.01
Female	28.6	184.5	27.1

AUC_(0–24h),_ area under the concentration–time curve.

### 3.2 Pharmacokinetic profile of X842 in dogs

**FIGURE 6:**
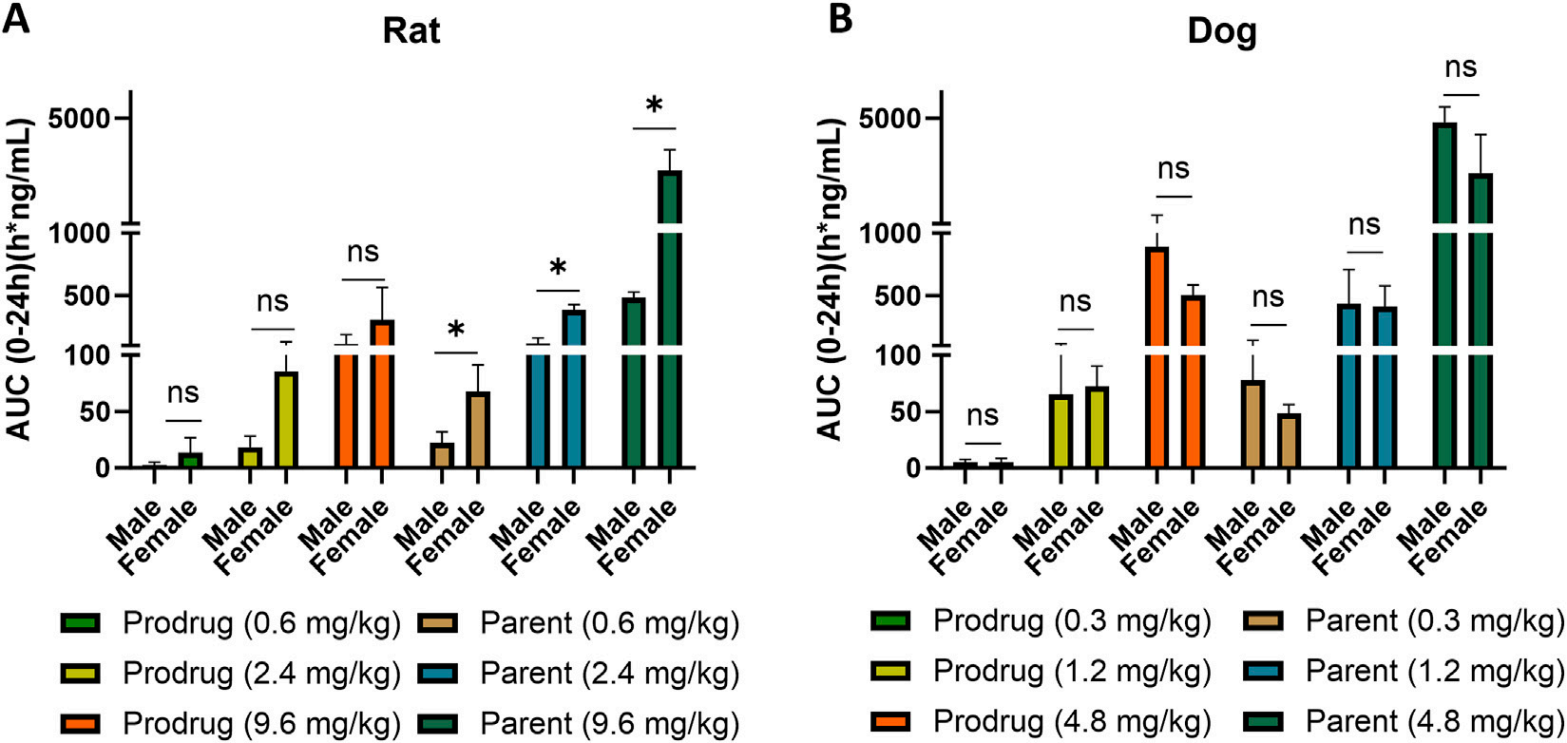
Comparison of X842 absorption [AUC_(0–24h)_] between male and female rats and dogs. Data from both rats **(A)** and dogs **(B)** are summarized. *P < 0.05, compared to the same parameter in males. AUC_(0–24h),_ area under the concentration–time curve.

In dogs, X842 was evaluated after IV, single oral, and repeated oral dosing. Following IV administration (Supplementary Table S1), t_1/2_ was short and was similar between the sexes (∼0.6 h), indicating rapid clearance. After a single oral dose (Supplementary Table S2), t_1/2_ increased to 2.0–3.4 h in males and to 2.1–3.2 h in females, consistent with rapid first-pass conversion of X842 to linaprazan. Unlike in rats, where females displayed significantly higher C_max_ and AUC_0–24h_ than males, oral exposure in dogs showed no sex-related differences (Supplementary Tables S2, S3). Across species, AUC_0–24h_ increased with the dose in both rats and dogs (Figure 6). In dogs, the increase from 1.2 to 4.8 mg/kg reached statistical significance (*P* < 0.05), confirming dose proportionality. Where female rats exhibited markedly higher parent drug exposure (*P* < 0.05), female dogs showed only a non-significant trend toward lower AUC_0–24h_ at the highest dose. Collectively, these data indicate a clear dose–response relationship in dogs and an absence of sex differences in oral absorption.

### 3.3 Efficacy of X842 on the activity of H^+^/K^+^-ATPase

**FIGURE 7:**
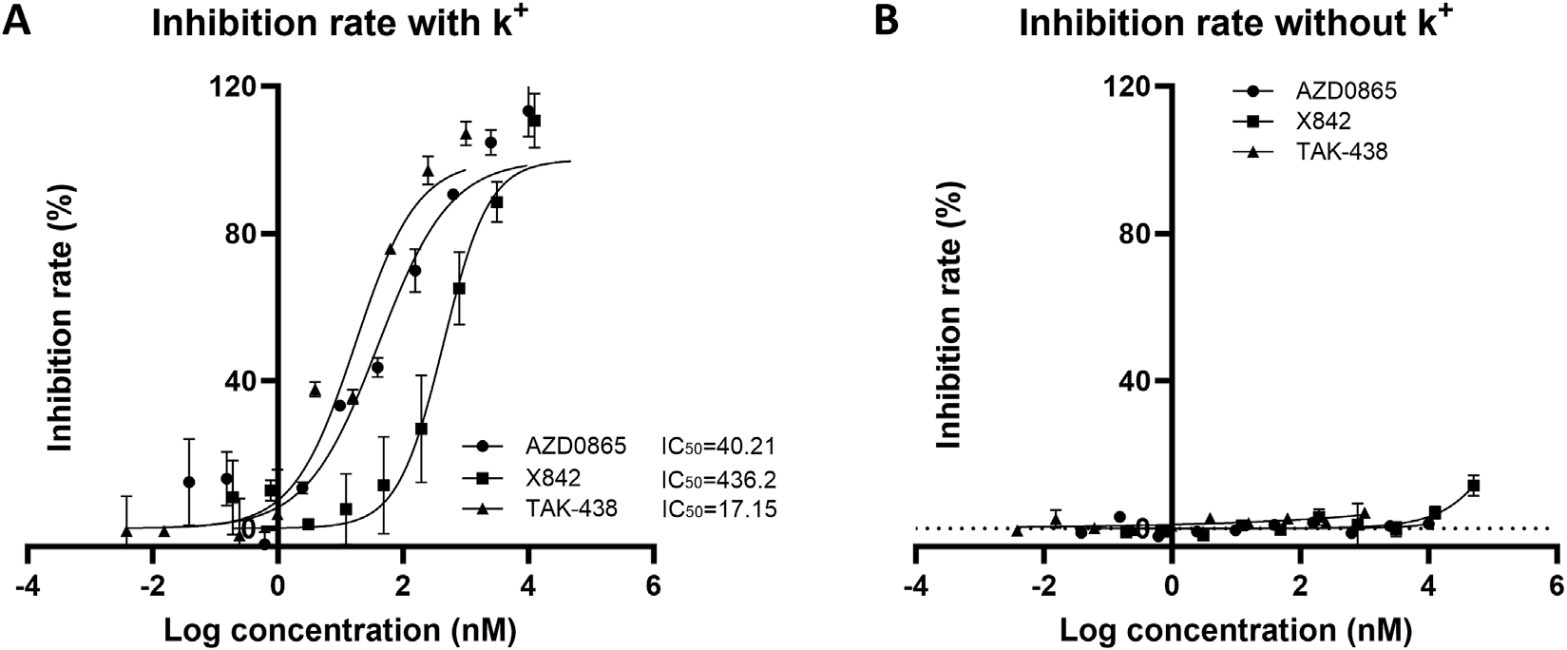
Inhibitory activity of H^+^/K^+^ ATPase from rabbit gastric glands by X842, linaprazan, and vonoprazan in the presence **(A)** and absence **(B)** of potassium ions. The data are presented as the mean ± standard error of three experiments.

After evaluating the pharmacokinetic profile of X842, its pharmacodynamic profile was investigated. The effect of X842 on H^+^/K^+^-ATPase activity was analyzed using an MLG-based assay, an indirect colorimetric method in which the readout has been cross-validated against radiometric ATP hydrolysis assays (Bandhuvula et al., 2007; Baykov et al., 2021) with correlation coefficients of >0.98, confirming its reliability for quantifying H^+^/K^+^-ATPase inhibition. The ATPase activity was not directly measured in this study because the inhibitory effects were quantified by calculating the percentage inhibition relative to the control wells. In the presence of K^+^, linaprazan and X842 inhibited H^+^/K^+^-ATPase activity in a concentration-dependent manner. The inhibitory potency of these three compounds ranked as follows: vonoprazan > linaprazan > X842. Their IC_50_ values were 17.15 nM (95% confidence interval [CI] 10.81–26.87 nM), 40.21 nM (95% CI 24.02–66.49 nM), and 436.20 nM (95% CI 227.3–806.6 nM), respectively (Figure 7). No measurable inhibitory effect was observed in the absence of K^+^. Compared with linaprazan, X842 exhibited nearly 10-fold lower inhibitory activity against H^+^/K^+^-ATPase in the presence of K^+^, which may be partially due to structural modifications.

### 3.4 Efficacy of X842 on gastric acid secretion in a pylorus-ligated rat model

In the pylorus-ligated rat model, we systematically compared the antisecretory potency of X842 with that of the positive control vonoprazan under conditions of basal acid secretion following simple pyloric ligation. Vonoprazan (2 mg/kg) suppressed total gastric acid by 47% relative to vehicle-treated controls (*P* < 0.05). Under identical conditions, X842 produced clear dose-dependent inhibition; 1.5, 1.0, and 0.5 mg/kg reduced acid by 85%, 61%, and 44%, respectively (all *P* < 0.05), whereas 0.15 mg/kg X842 had no significant effect (6% inhibition, *P* > 0.05) (Table 5). The dose required for 50% inhibition (ID_50_) was 0.55 mg/kg for X842, substantially lower than the dose of 2 mg/kg vonoprazan. X842 administered at ≥1 mg/kg consistently outperformed vonoprazan at 2 mg/kg in terms of both absolute acid reduction and fractional inhibition. Collectively, this demonstrated that X842 was a more potent acid suppressant than vonoprazan in this validated preclinical model, as well as evidencing its therapeutic potential in the histamine-stimulated pylorus-ligation model (Supplementary Figure S3; Supplementary Table S4).

**TABLE 5:** Total rate of acid inhibition by X842 in pylorus-ligated rats.

Group	Total acidity inhibitory rate (%)
Vehicle	-
Vonoprazan (positive control)	47
1.5 mg/kg	85
1.0 mg/kg	61
0.5 mg/kg	44
0.15 mg/kg	6

## 4 Discussion

Linaprazan, an H^+^/K^+^-ATPase inhibitor, effectively inhibits gastric acid secretion, both in animals and humans, exhibiting a fast onset and acceptable tolerability (Holstein et al., 2004; Leowattana and Leowattana, 2022). However, linaprazan has failed to demonstrate superior efficacy to existing PPIs, especially regarding long-term use and control of nocturnal acid breakthrough (Dent et al., 2008).

To address these pharmacokinetic limitations, a series of linaprazan prodrugs were synthesized. The lead candidate, linaprazan glurate, was selected for detailed evaluation in the present study. Its modification via glurate acid esterification attenuates the rapid release of linaprazan, minimizes plasma fluctuation, and creates a protracted pharmacokinetic profile that enhances gastric tissue retention (tissue-to-plasma AUC_0–48h_ 13.03–17.94 in rats (Zhang et al., 2024)). Critically, unpublished Phase I data confirm sustained acid suppression with X842. The oral administration of X842 demonstrated dose-dependent acid suppression efficacy. Crucially, ≥50 mg doses achieved gastric pH ≥4 holding time ratio (HTR) >50% throughout the 24-h period, with nocturnal efficacy specifically evidenced by ≥100 mg doses maintaining pH ≥4 HTR >50% during nocturnal periods (12–24 h), translating to significant nocturnal acid breakthrough (NAB) mitigation. Its 24-h pH control is designed to overcome nocturnal acid breakthrough, while its reversible K^+^-competitive mechanism remains fully active in the absence of acid activation, offering an effective alternative for patients who are refractory to PPIs. X842 thus represents a promising candidate for severe acid-related disorders. Therefore, X842 has the potential to be further developed into one of the best drugs for the treatment of severe diseases related to gastric acid secretion.

### 4.1 Properties of X842 as a prodrug

**FIGURE 8:**
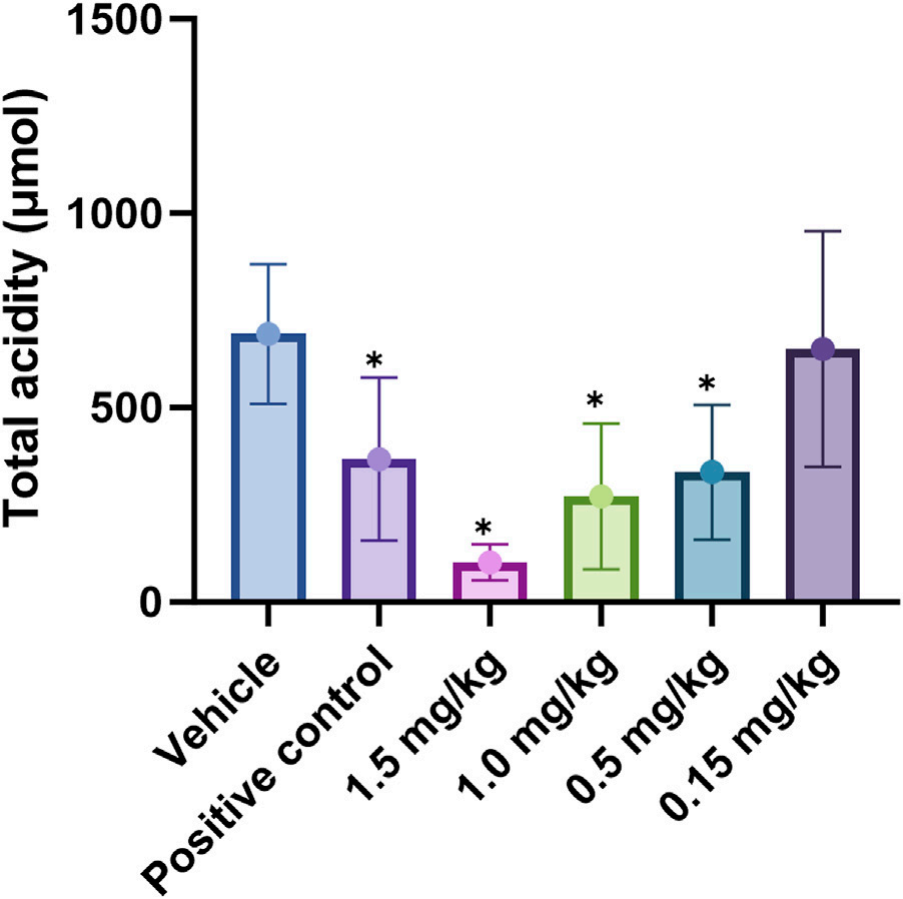
Effect of X842 on total acidity in a pylorus-ligated rat model. *P < 0.05, compared with the same parameter in the presence of the blank vehicle (independent-samples t-test). The effect of vonoprazan (positive control) was only tested at 2 mg/kg (N = 12 per group).

Linaprazan and vonoprazan are well characterized P-CABs (Gedda et al., 2007; Hori et al., 2011). In the present study, we compared the gastric acid inhibitory properties of linaprazan, vonoprazan, and X842. X842 showed significant inhibitory effects on H^+^/K^+^-ATPase in the presence of K^+^ compared with in the absence of K^+^ (Figure 7). The potency of X842 *in vitro* was 20-times lower than that of linaprazan and vonoprazan when cellular esterase was not present (i.e., when X842 had not been metabolized). However, *in vivo* experiments using a pylorus-ligated rat model (Figure 8) showed that X842 for inhibiting acid secretion was more potent and had a longer duration of action than vonoprazan. The prodrug nature of X842 was confirmed by two additional *in vivo* experiments. The acid inhibitory effects were exerted by the metabolites of X842. After administration to rats, the percentage of X842 that was transformed into linaprazan ranged from 48.69% to 59.48% (Table 2). After administration to dogs, the transformed percentage ranged from 34.23% to 57.02% (Supplementary Table S2). Thus, the desired pharmacological properties of X842 were achieved by its designation as a prodrug of linaprazan.

### 4.2 Pharmacological efficacy of X842, with dual actions on H^+^/K^+^-ATPase via the prodrug and its metabolites

Vonoprazan was a first-in-class P-CAB that entered the market with higher potency and longer-lasting acid suppression than conventional PPIs (Otake et al., 2016; Garnock-Jones, 2015). X842 surpassed vonoprazan *in vivo*. For instance, at doses of ≥1 mg/kg, it achieved a higher rate of acid suppression than 2 mg/kg vonoprazan (Figure 8). Moreover, even under non-metabolic conditions *in vitro*, X842 retained measurable activity against H^+^/K^+^-ATPase in the presence of K^+^, but it was 20-fold weaker than linaprazan or vonoprazan (Figure 7). This indicates that both the prodrug and its active metabolite contribute to the effect, a profile already hinted at in preclinical and phase I studies (data to be published) and characterized by rapid onset and prolonged action. Although these observations suggest a dual pharmacodynamic mechanism, we cannot rule out esterase-mediated conversion within the enzyme preparation. Experiments with purified enzyme systems or esterase inhibitors will be required for definitive confirmation. Collectively, the data show that X842 is a potent, fast-acting, and long-lasting H^+^/K^+^-ATPase inhibitor. Such properties position it as a promising therapeutic option for acid-related disorders, particularly in clinical settings where current PPI therapy remains inadequate.

### 4.3 Pharmacokinetic profile of X842

As described previously (Rawla et al., 2018), linaprazan was quickly absorbed with a relatively short t_1/2_, but it failed to achieve 24-h inhibition of gastric acid secretion. In the pharmacokinetic analysis, X842 reduced C_max_ and extended the t_1/2_ of linaprazan (Table 1; Table 2). As mentioned, X842 may have a dual pharmacodynamic action in which the prodrug acts on the H^+^/K^+^-ATPase in the stomach before being metabolized. This could be an advantage of X842, conferring both fast-onset and prolonged-action properties on the inhibition of gastric acid secretion after oral administration. Compared with linaprazan, another advantage of X842 was the reduced toxicity (data to be published). No significant adverse effects, including acute, reproductive, teratogenic, or carcinogenic toxicity, were observed in either rats or dogs. The prodrug X842 minimizes hepatic exposure to linaprazan owing to its two-compartment pharmacokinetics, which also effectively minimizes toxicity.

As a novel P-CAB, the structural features of X842 in terms of the ester component are designed to influence its pharmacokinetic properties. *In vivo*, the prodrug is rapidly hydrolyzed by carboxylesterase 2 to yield the active metabolite linaprazan (for detailed experimental evidence see our companion study), which subsequently undergoes oxidation, dehydrogenation, and glucuronidation (Zhang et al., 2024). We noted that the plasma concentrations of both X842 and its metabolite linaprazan in female rats were higher than in male rats (Figures 4, 5; Table 2). This sex difference was not observed after IV administration, suggesting differences in the absorption and metabolism of X842 in rodents after oral administration. In fact, imidazopyridine derivatives are major inhibitors of cytochrome P450 (CYP) 3A4 enzymes (Martignoni et al., 2006). The CYP3A subfamily of isoforms differ between the sexes in rats (Song et al., 2012). However, in contrast to the results seen in rats, no sex differences in the absorption or plasma concentrations of X842 or linaprazan were observed in dogs (Supplementary Table S2; Figure 6). Although differences in drug absorption and metabolism among animal species are not uncommon, these observed species differences should be considered in further studies in humans. However, no sex differences have been observed in phase I trials involving more than 80 participants with equal numbers of both men and women (data to be published) after oral administration of X842. The consistency between the pharmacokinetic characteristics of X842 and linaprazan in humans and dogs is understandable, as CYP polymorphisms in humans and canines are much more closely related to each other than to rodents (Song et al., 2012).

## 5 Conclusion

To address the persistent unmet medical needs of acid-related disorders such as GERD, X842, a prodrug of linaprazan, was studied for its pharmacological and pharmacokinetic profiles *in vitro* and *in vivo*. X842 exhibited a pronounced inhibitory effect on H^+^/K^+^-ATPase *in vitro* and required K^+^ for this effect. It also effectively inhibited gastric acid secretion in rat models of gastric acid secretion. Second, the prodrug had improved pharmacokinetic and pharmacological profiles compared with the parent drug by reducing acute C_max_ and extending t_1/2_. X842 has both a rapid onset and a long duration of action on acid inhibition in animal models. Additionally, compared with vonoprazan, X842 demonstrated a 30% lower effective dose (ID_50_ 0.55 mg/kg, Figure 8) in the same rat model and achieved at least 24 h of acid suppression after a single oral dose, whereas vonoprazan required twice-daily dosing to maintain a similar pH-holding time. Collectively, these data position X842 as the best-in-class P-CAB for severe acid-related disorders.

## Data Availability

The raw data supporting the conclusions of this article will be made available by the authors, without undue reservation.
