# Supplementary material for: Pharmacological characterization of linaprazan glurate (X842), a novel potassium-competitive acid blocker, in vitro and in vivo
Source: Front Pharmacol. 2025 Sep 5;16:1636523. doi: 10.3389/fphar.2025.1636523 (PMC12446003; doi:10.3389/fphar.2025.1636523)
Supplement: Supplementary file 1 [file DataSheet1.pdf]

Pharmacological characterization of linaprazan glurate (X842), a novel potassium-competitive acid blocker, in vitro and in vivo

Ming Lu[a#], Yi Cui[b#], Nailin Li[c], Yan He[d], Ling Zhou[a], Jin Xiu[a,b,e], Pingsheng Hu[a,b*]

a Sinorda Pharmaceuticals Ltd., Jing Yang Hi-Tech Park, Guiyang, Guizhou, P. R. China

b Clinical Research Center, The Affiliated Hospital of Guizhou Medical University, Guiyang, Guizhou, P. R. China

c Clinical Pharmacology Group, Department of Medicine-Solna, Karolinska University Hospital-Solna, Karolinska Institutet, Stockholm, Sweden

d Good Clinical Practice Center, The Affiliated Hospital of Guizhou Medical University, Guiyang, Guizhou, P. R. China

e Department of Generic, Karolinska Institutet, Department of Neurobiology, Care Sciences and Society, Division of Clinical Geriatrics, Stockholm, Sweden

#Ming Lu and Yi Cui contributed equally to this work.

* Corresponding authors.

Supplementary Material

1. Methods

1.1 Histamine-stimulated acid secretion in pylorus-ligated rat models

The rats were fasted overnight with ad libitum access to water. Vehicle or X842 (2 mg/kg, 8 mg/kg) was administered orally to male Sprague-Dawley rats using a feeding catheter in a blinded manner. One hour after dosing, the pylorus was ligated under isoflurane anesthesia, and histamine (30 mg/kg/10 mL) was immediately administered subcutaneously. At 3 hours after histamine administration, the rats were sacrificed by carbon dioxide asphyxiation. The gastric contents were collected from the removed stomach. The gastric contents were collected and centrifuged at 3000 rpm for 10 minutes. The volume of each sample was measured, the gastric acid concentration was determined by acid-base titration, and the total acidity was calculated.

2. Supplementary Figures and Tables

2.1 Supplementary Figures

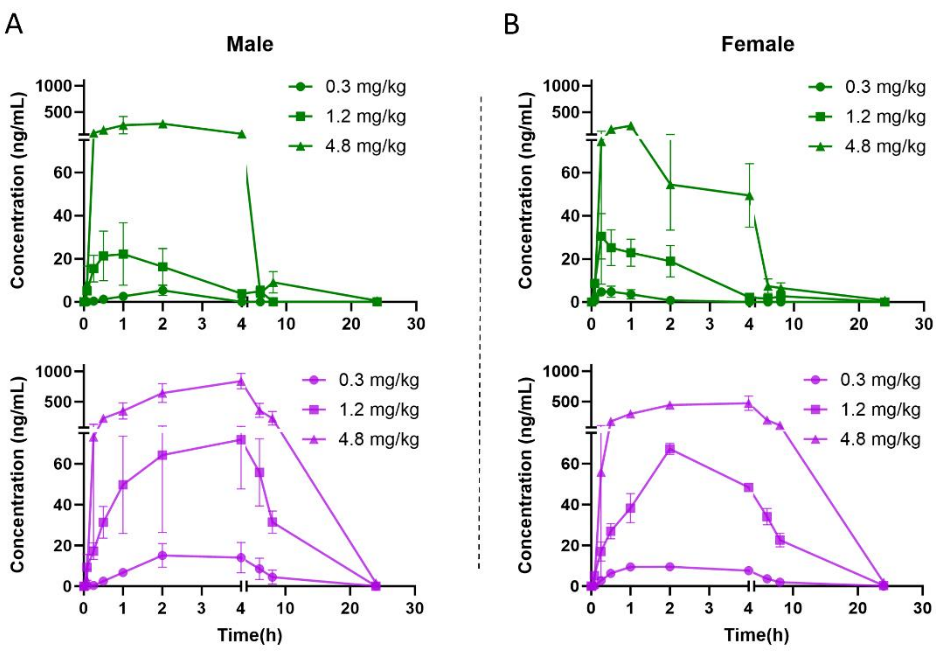

Figure S1. Plasma concentration of X842 after oral administration in male (A) and female (B) dogs. Both the prodrug form (X842) and the parent form (linaprazan) were evaluated (N=3 per group).

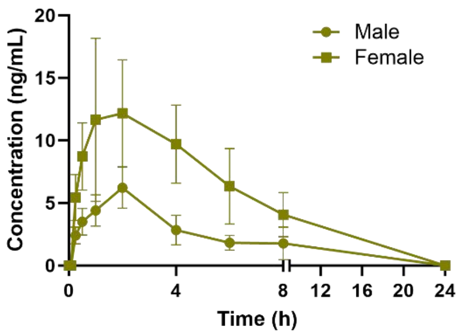

Figure S2. Plasma concentration of linaprazan (the parent form of X842) after oral administration of X842 in male and female rats at 0.6 mg/kg.

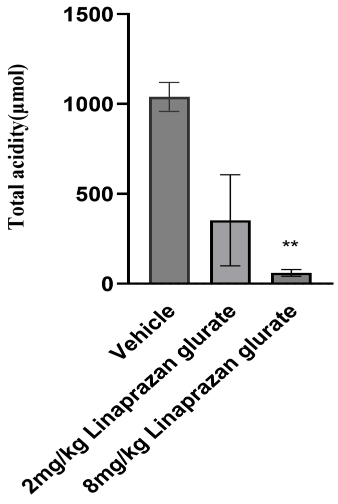

Figure S3. Effect of linaprazan glurate (X842) on total acidity under histamine stimulation in pylorus-ligated rats (experimental details: Supplementary Method 1.1).
**P < 0.01 vs. blank vehicle control (independent-samples t-test). Vehicle control: n = 3, X842 2 mg/kg: n = 2, X842 10 mg/kg: n = 3.

2.2. Supplementary Tables

Table S1. Pharmacokinetic parameters of X842 after intravenous administration in male and female dogs (0.3 mg/kg)

Parameter		Male		Female	
		Prodrug	Parent	Prodrug	Parent
$t_{1/2}$	h	0.5±0.1	1.5±0.25	0.7±0.1	1.6±0.2
C_{max}	ng/mL	744.9±156.8	45.5±21.4	693.8±224.8	30.6±6.2
$AUC_{(0\text{-}24h)}$	h*ng/mL	361.0±118.5	170.4±26.2	343.2±125.3	106.4±13.9

$AUC_{(0\text{-}24h)}$, area under the concentration time curve; C_{max}, maximum concentration; $t_{1/2}$, half-life.

Table S2. Pharmacokinetic parameters of X842 after oral administration in male and female dogs

Parameter	Unit	0.3 mg/kg		1.2 mg/kg		4.8 mg/kg	
		Prodrug	Parent	Prodrug	Parent	Prodrug	Parent
Male							
$t_{1/2}$	h	NA	2.2±0.7	2.0±0.7	2.8±0.2	3.4±2.2	2.6±0.2
C_{max}	ng/mL	5.3±4.0	15.8±11.24	26.3±21.6	82.8±56.5	355.3±211.8	851.0±116.1
$AUC_{(0\text{-}24h)}$	h*ng/mL	5.2±2.3	77.8±66.8	65.1±51.2	436.5±272.2	889.4±434.7	4845.9±592.9*
Female							
$t_{1/2}$	h	NA	2.1±0.4	1.2±0.2	3.2±0.42	2.7±2.2	2.2±0.9

C_{max}	ng/mL	6.1±1.46	10.4±1.4	37.5±7.6	67.2±4.7	271.3±52.9	534.8±148.6
$AUC_{(0-24h)}$	h*ng/mL	5.1±3.3	48.7±7.4	72.3±18.3	412.1±165.6	503.8±82.8	2919.4±1472.8

$AUC_{(0-24h)}$, area under the concentration time curve; C_{max}, maximum concentration; $t_{1/2}$, half-life.

Table S3. Accumulation of X842 after 7 days of repeat oral administration in dogs (N=3 per group)

Parameter	Unit	Day 1		Day 7	
		Prodrug	Parent	Prodrug	Parent
		Male			
$t_{1/2}$	h	2.01±0.73	2.8±0.2	8.6±10.8	3.8±0.4
C_{max}	ng/mL	26.3±21.57	82.8±56.5	15.6±10.4	67.5±23.9
$AUC_{(0-24h)}$	h*ng/mL	65.1±51.23	436.5±272.2	35.4±8.7	439.3±81.6
Accumulation coefficient	-	0.8	1.2	-	-
		Female			
$t_{1/2}$	h	1.1±0.2	3.2±0.42	2.0±1.2	5.1±1.1
C_{max}	ng/mL	137.5±7.6	67.2±4.7	47.2±10.6	65.0±4.7
$AUC_{(0-24h)}$	h*ng/mL	72.3±18.3	412.1±165.6	69.4±54.1	508.3±41.8
Accumulation coefficient	-	0.9	1.3	-	-

Accumulation coefficient = $AUC_{(0-24h)}$ (Day 7 of repeat administration)/$AUC_{(0-24h)}$ (Day 1 of repeat administration)

$AUC_{(0-24h)}$, area under the concentration time curve; C_{max}, maximum concentration; $t_{1/2}$, half-life.

Table S4. Total inhibitory rate of X842 on histamine-stimulated acid secretion in pylorus-ligated rats (experimental details: Supplementary Method 1.1).

Group	Total acidity inhibitory rate (%)
Vehicle	-
2 mg/kg linaprazan glurate	66
8 mg/kg linaprazan glurate	71
